# Supplementary material for: VivosX, a disulfide crosslinking method to capture site-specific, protein-protein interactions in yeast and human cells
Source: eLife. 2018 Aug 9;7:e36654. doi: 10.7554/eLife.36654 (PMC6107336; doi:10.7554/eLife.36654)
Supplement: Supplementary file 1. [file elife-36654-supp1.docx]

| **Strain** | **Genotype** | **Source/ Reference** |
| --- | --- | --- |
| *BY4741* | *MAT a his3∆1 leu2∆0 met15∆0 ura3∆0* | *Standard strain* |
| *yEL242* | *BY4741 htz1::HTZ1-2xFLAG-URA3* | *This study* |
| *yEL243* | *BY4741 htz1::htz1(H45C)-2xFLAG-URA3* | *This study* |
| *yEL244* | *BY4741 htz1::htz1(A46C)-2xFLAG-URA3* | *This study* |
| *yEL245* | *BY4741 htz1::htz1(T47C)-2xFLAG-URA3* | *This study* |
| *yEL246* | *BY4741 htz1::htz1(G48C)-2xFLAG-URA3* | *This study* |
| *yEL247* | *BY4741 htz1::htz1(R49C)-2xFLAG-URA3* | *This study* |
| *yEL248* | *BY4741 htz1::htz1(T50C)-2xFLAG-URA3* | *This study* |
| *yEL272* | *BY4741 htz1::htz1(A46C,G48C)-2xFLAG-URA3 YBR209w_5'::rxYFP-NLS-LEU2* | *This study* |
| *yEL273 (FY406)* | *MAT a his3∆200, trp1∆63, lys2-128δ, ura3-52, and leu2∆1 (hta1-htb1)∆::LEU2 (hta2-htb2)∆::TRP1 [URA3 CEN ARS HTA-HTB1]* | *Hirschhorn et al. MCB 1995 v15 p1999* |
| *yEL284* | *MAT a his3∆200, trp1∆63, lys2-128δ, ura3-52, and leu2∆1 (hta1-htb1)∆::LEU2 (hta2-htb2)∆::TRP1 [HIS3 CEN ARS HTA-HTB1]* | *This study* |
| *yEL285* | *MAT a his3∆200, trp1∆63, lys2-128δ, ura3-52, and leu2∆1 (hta1-htb1)∆::LEU2 (hta2-htb2)∆::TRP1[HIS3 CEN ARS 2xV5-HTA-HTB1]* | *This study* |
| *yEL286* | *MAT a his3∆200, trp1∆63, lys2-128δ, ura3-52, and leu2∆1 (hta1-htb1)∆::LEU2 (hta2-htb2)∆::TRP1 [HIS3 CEN ARS 2xV5-HTA1(N40C)-HTB1]* | *This study* |
| *yEL489* | *MAT a his3∆200, trp1∆63, lys2-128δ, ura3-52, and leu2∆1 (hta1-htb1)∆::LEU2 (hta2-htb2)∆::TRP1 [HIS3 CEN ARS 2xV5-HTA1(G39C)-HTB1]* | *This study* |
| *yEL490* | *MAT a his3∆200, trp1∆63, lys2-128δ, ura3-52, and leu2∆1 (hta1-htb1)∆::LEU2 (hta2-htb2)∆::TRP1 [HIS3 CEN ARS 2xV5-HTA1(Y41C)-HTB1]* | *This study* |
| *yEL491* | *MAT a his3∆200, trp1∆63, lys2-128δ, ura3-52, and leu2∆1 (hta1-htb1)∆::LEU2 (hta2-htb2)∆::TRP1 [HIS3 CEN ARS 2xV5-hta1(A42C)-HTB1]* | *This study* |
| *yEL492* | *MAT a his3∆200, trp1∆63, lys2-128δ, ura3-52, and leu2∆1 (hta1-htb1)∆::LEU2 (hta2-htb2)∆::TRP1 [HIS3 CEN ARS 2xV5-HTA1(Q43C)-HTB1]* | *This study* |
| *yEL274* | *BY4741 swc5∆::kanMX* | *Open biosystems* |
| *yEL400* | *BY4741 swc5∆::kanMX htz1::htz1(T47C)2xFL* | *This study* |
| *yEL422* | *BY4741 swc5∆::kanMX htz1::htz1(T47C)2xFL [URA3 CEN ARS]* | *This study* |
| *yEL423* | *BY4741 swc5∆::kanMX htz1::htz1(T47C)2xFL [URA3 CEN ARS SWC5]* | *This study* |
| *yEL424* | *BY4741 swc5∆::kanMX htz1::htz1(T47C)2xFL [URA3 CEN ARS swc5(1-232)]* | *This study* |
| *yEL425* | *BY4741 swc5∆::kanMX htz1::htz1(T47C)2xFL [URA3 CEN ARS swc5(79-303)]* | *This study* |
| *yEL426* | *BY4741 swc5∆::kanMX htz1::htz1(T47C)2xFL [URA3 CEN ARS swc5(LDW --> 3A)]* | *This study* |
| *yEL044 (HHY221)* | *W303 MAT a tor1-1 fpr1::P-LEU2-P RPL13A-2XFKBP12::P* | *Laemmli(2008)MolCell* |
| *yEL401* | *W303 MAT a tor1-1 fpr1::P-LEU2-P RPL13A-2XFKBP12::P htz1::htz1(T47C)2xFL-URA3* | *This study* |
| *yEL054* | *W303 MAT a tor1-1 fpr1::P-LEU2-P RPL13A-2XFKBP12::P swc5::SWC5-FRB-HISMX6* | *This study* |
| *yEL403* | *MAT a tor1-1 fpr1::LEU2 RPL13A-FK::P SWC5-FRB::kanMX6 htz1::htz1(T47C)2xFL-URA3* | *This study* |
| *yEL098* | *W303 MAT a tor1-1 fpr1::LEU2 RPL13A-2XFKBP::P spt15::SPT15-FRB-GFP-kanMX6* | *Tramantano et al. 2016 eLIFE* |
| *yEL402* | *MAT a tor1-1 fpr1::LEU2 RPL13A-FK::P SPT15-FRB-GFP::kanMX6 htz1::htz1(T47C)2xFL-URA3* | *This study* |
| *yEL311* | *MAT a his3∆200, trp1∆63, lys2-128δ, ura3-52, and leu2∆1 (hta1-htb1)∆::LEU2 (hta2-htb2)∆::TRP1 htz1::HTZ1-2xFL-URA3 [HIS3 CEN ARS 2xV5-HTA1-HTB1]* | *This study* |
| *yEL314* | *MAT a his3∆200, trp1∆63, lys2-128δ, ura3-52, and leu2∆1 (hta1-htb1)∆::LEU2 (hta2-htb2)∆::TRP1 htz1::HTZ1-2xFL-URA3 [HIS3 CEN ARS 2xV5-hta1(N39C)-HTB1]* | *This study* |
| *yEL349* | *MAT a his3∆200, trp1∆63, lys2-128δ, ura3-52, and leu2∆1 (hta1-htb1)∆::LEU2 (hta2-htb2)∆::TRP1 htz1::htz1(T46C)-2xFL-URA3 [HIS3 CEN ARS 2xV5-hta1(N39C)-HTB1]* | *This study* |
| *yEL356* | *MAT a his3∆200, trp1∆63, lys2-128δ, ura3-52, and leu2∆1 (hta1-htb1)∆::LEU2 (hta2-htb2)∆::TRP1 htz1::htz1(T46C)-2xFL-URA3 [HIS3 CEN ARS 2xV5-HTA1-HTB1]* | *This study* |
