# Supplementary material for: VivosX, a disulfide crosslinking method to capture site-specific, protein-protein interactions in yeast and human cells
Source: eLife. 2018 Aug 9;7:e36654. doi: 10.7554/eLife.36654 (PMC6107336; doi:10.7554/eLife.36654)
Supplement: Supplementary file 2. [file elife-36654-supp2.docx]

| PLASMID | DESCRIPTION | SOURCE |
| --- | --- | --- |
| *pRS416* | *URA3 CEN ARS* | *Sikorski and Hieter. Genetics (1989)* |
| *pEL353* | *URA3 CEN ARS-HTZ1-2FL* | *X. Shen* |
| *pEL427* | *URA3 CEN ARS-HTZ1-H44C-2FL* | *This study* |
| *pEL428* | *URA3 CEN ARS-HTZ1-G47C-2FL* | *This study* |
| *pEL429* | *URA3 CEN ARS-HTZ1-R48C-2FL* | *This study* |
| *pEL430* | *URA3 CEN ARS-HTZ1-T49C-2FL* | *This study* |
| *pEL431* | *URA3 CEN ARS-HTZ1-A45C-2FL* | *This study* |
| *pEL432* | *URA3 CEN ARS-HTZ1-T46C-2FL* | *This study* |
| *pSAB6* | *URA3 CEN ARS HTA1-HTB1* | *Hirschhorn et al. MCB (1995)* |
| *pEL299 (pJH55)* | *HIS3 CEN ARS HTA1-HTB1* | *Hirschhorn et al. MCB (1995)* |
| *pEL305* | *HIS3 CEN ARS 2xV5-HTA-HTB1* | *This study* |
| *pEL558* | *HIS3 CEN ARS-2xV5-HTA1(G39C)-HTB1* | *This study* |
| *pEL440* | *HIS3 CEN ARS-2xV5-HTA1(N40C)-HTB1* | *This study* |
| *pEL559* | *HIS3 CEN ARS-2xV5-HTA1(Y41C)-HTB1* | *This study* |
| *pEL560* | *HIS3 CEN ARS-2xV5-HTA1(A42C)-HTB1* | *This study* |
| *pEL561* | *HIS3 CEN ARS-2xV5-HTA1(Q43C)-HTB1* | *This study* |
| *pEL460* | *URA3 CEN ARS-SWC5* | *Sun et. al 2017 NAR* |
| *pEL468* | *URA3 CEN ARS-swc5(1-232)* | *Sun et. al 2017 NAR* |
| *pEL477* | *URA3 CEN ARS-swc5(79-303)* | *Sun et. al 2017 NAR* |
| *pEL479* | *URA3 CEN ARS-swc5(LDW --> 3A)* | *Sun et. al 2017 NAR* |
|  | | |
| LENTIVIRAL VECTOR | | SOURCE |
| *pBABE-hygro-rtTA* | | *Leung and Brugge. Nature (2012)* |
| *pLT-iGSP* | | *Leung and Brugge. Nature (2012)* |
| *pLT-H2AFZ-V5-iGSP* | | *This study* |
| *pLT-H2AFZ(H43C)-V5-iGSP* | | *This study* |
| *pLT-H2AFZ(H43C-K120R-K121R)-V5-iGSP* | | *This study* |
| *pLT-H2AFZ(K120R-K121R)-V5-iGSP* | | *This study* |
